# Genome-Wide Identification and Characterization of Actin-Depolymerizing Factor (*ADF*) Family Genes and Expression Analysis of Responses to Various Stresses in *Zea Mays* L.

**DOI:** 10.3390/ijms21051751

**Published:** 2020-03-04

**Authors:** Jun Huang, Wei Sun, Jiaxin Ren, Ruichun Yang, Jingsheng Fan, Yunfeng Li, Xin Wang, Shija Joseph, Wenbin Deng, Lihong Zhai

**Affiliations:** 1 The State Key Laboratory for Conservation and Utilization of Subtropical Agro-bioresources, South China Agricultural University, Guangzhou 510642, China; junhuang@scau.edu.cn (J.H.); rchyang@scau.edu.cn (R.Y.); jingshengfan@yeah.net (J.F.); liyunfeng2019@stu.scau.edu.cn (Y.L.); wangxin2019@stu.scau.edu.cn (X.W.); 2 College of Agriculture and Biology, Zhongkai University of Agriculture and Engineering, Guangzhou 510225, China; Starking521@webmail.hzau.edu.cn; 3 Medical College, Hubei University of Arts and Science, Xiangyang 441053, China; 2017140251@hbuas.edu.cn (J.R.); Josephsimon378@gmail.com (S.J.); wbdeng@ucdavis.edu (W.D.)

**Keywords:** *Zea Mays* L., *ADF* genes, abiotic stresses, expression pattern

## Abstract

Actin-depolymerizing factor (ADF) is a small class of actin-binding proteins that regulates the dynamics of actin in cells. Moreover, it is well known that the plant ADF family plays key roles in growth, development and defense-related functions. Results: Thirteen maize (*Zea mays* L., ZmADFs) ADF genes were identified using Hidden Markov Model. Phylogenetic analysis indicated that the 36 identified ADF genes in *Physcomitrella patens*, *Arabidopsis thaliana, Oryza sativa japonica,* and *Zea mays* were clustered into five groups. Four pairs of segmental genes were found in the maize ADF gene family. The tissue-specific expression of ZmADFs and OsADFs was analyzed using microarray data obtained from the Maize and Rice eFP Browsers. Five ZmADFs (*ZmADF1/2/7/12/13*) from group V exhibited specifically high expression in tassel, pollen, and anther. The expression patterns of 13 ZmADFs in seedlings under five abiotic stresses were analyzed using qRT-PCR, and we found that the ADFs mainly responded to heat, salt, drought, and ABA. Conclusions: In our study, we identified ADF genes in maize and analyzed the gene structure and phylogenetic relationships. The results of expression analysis demonstrated that the expression level of ADF genes was diverse in various tissues and different stimuli, including abiotic and phytohormone stresses, indicating their different roles in plant growth, development, and response to external stimulus. This report extends our knowledge to understand the function of ADF genes in maize.

## 1. Introduction

In eukaryotes, actin-depolymerizing factor (ADF) is conserved and performs an essential function in actin dynamics which is important for cell motility, development, differentiation, signal transduction cytokinesis, and maintenance of eukaryotic cell surface structure [1,2,3]. ADF was first isolated from the porcine brain and named “cofilin,” meaning “cofilamentous protein” [4,5]. As a large ubiquitous family, ADFs are of low molecular mass ranging from 15 to 22 kD [6], and in order to improve their activity of depolymerization into actin monomers (G-actin), ADFs always bind to actin filaments (F-actin) [1]. There are only one or two ADF/cofilin genes in the genome of most non-plant organisms, whereas a large ADF gene family is formed in most plants, resulting in the differentiation of physiological functions between non-plant and plant organisms. Currently, the identification of whole-genome ADF gene family in many plants has been reported. For instance, there were 11 ADF genes in rice [7], 11 in *Arabidopsis* [8], 27 in banana [9], 14 in poplar [10], and 11 in tomato [11]. The phylogenetic analysis and expression profiling of higher-plant ADFs, such as *Arabidopsis*, rice, and tomato, has been reported, but not with the maize ADF gene family. Generally, according to phylogenetic analysis of their amino acid sequences, the ADF protein in higher plant can be divided into four subclasses (I–IV) [7]. *AtADF1*, *-2*, *-3*, and *-4* comprise subclass I and are expressed at a relatively high level throughout the plant except pollen, whereas *AtADF7* and *AtADF10* which belong to subclass IIa exhibit pollen-specific expression patterns. *AtADF8* and *AtADF11* (IIb) are expressed specifically in root epidermal cells. Subclass III (*AtADF5* and *AtADF9*) and subclass IV (*AtADF6*) exhibit universal expression in different tissues, but *AtADF9* is more strongly expressed in root subapical region, trichomes, shoot apical meristem (SAM), and callus [8]. The expression of *AtADF5* is limited to the root tip meristem [12].

Currently, there are only a few reports on the analysis of plant ADF genes. The ADF gene family is well known to make a special contribution to the processes of plant growth and stress responses, including pollen tube development [12,13,14,15,16], cotton fiber development [17,18], root hairs and trichomes (leaf hairs) morphogenesis [14], pathogen defense [19,20,21,22,23], drought tolerance [24], etc. For example, knockdown or overexpression of *AtADF1* significantly elongates or shortens organ length, such as that of roots, hypocotyls, cotyledons, and stems. Moreover, downregulation of *AtADF1* expression significantly delays flowering time, without affecting that in overexpressing plants [25,26]. *GhADF1* is a constitutive expression gene in cotton, while abundantly in fiber cells, and the downregulation of *GhADF1* alters the cell wall morphology and elongates the length of fiber cells [17,18]. Wavy mature leaves have been observed in *AtADF2* and *AtADF4* knockdown lines [19]. In addition, loss of *AtADF4* causes hypocotyl elongation under dark growth condition [27], whereas it leads to root hairs shortening under the standard growth condition with light [28]. Recently, the knockout mutant of *AtADF4* shows significantly enhanced resistance against powdery mildew fungus [28], while *AtADF2* knockdown leads to cellular defects but successfully prevents nematode infection [19]. The phloem expressed *AtADF3* is required for positively controlling GPA (green peach aphid) infestation [29]. Therefore, subclass I ADFs have the function of maintaining the normal growth of the plant and conferring resistance to various abiotic/biotic stresses.

During pollen germination and pollen tube growth, *AtADF5*, but not *AtADF9*, makes an important impact in the formation, organization, and maintenance of actin bundles [30]. The loss-function mutant of pollen specifically expresses *AtADF7* which significantly suppresses the pollen tube growth [31] and *AtADF10* is a pollen-specific ADF, however, *AtADF7* and *AtADF10* show different spatial profiles, indicating their unique functions in the pollen tube for regulating actin dynamics [16]. The expression of *AtADF9* in the root tip meristem, trichomes, and SAM is increased by 2,4-D, IAA, kinetin, GA3, and ABA, and the knockout mutant of *AtADF9* shows early flowering and small plant size under long-day conditions [14].

*TaADF3* is induced by ABA, drought and cold, and negatively regulated wheat resistance to *Puccinia striiformis f.sp.tritici* (*Pst*) [22]. Whereas *OsADF3* is reduced by salt stress and induced by exogenous ABA and cold, and *OsADF3*-transgenic *Arabidopsis* shows increased drought stress tolerance [24]. *TaADF4* is also upregulated by JA and ABA, but differs from *TaADF3*, which positively modulates wheat resistance to *Pst* [23]. Similar to *TaADF4*, *TaADF7* also positively contributes to wheat resistance to *Pst* [21]. The TaADF is also required for cold tolerance in wheat [32].

In maize, three ZmADFs (ZmABPs, then renamed as ZmADFs) have been cloned and their functions have been discovered. Similar to *LiADF1* [15], *ZmADF1/2* are expressed only in pollen which function in pollen actin reorganization [13,33], whereas *ZmADF3* is expressed in every examined maize tissue except pollen which is a vegetative gene and, furthermore, *ZmADF3* binds quantitatively to both monomeric and polymeric [34]. However, the entire ADF family in the maize genome has not been systematically identified, and expression in different organs and the abiotic stresses response are unrevealed. In our study, we performed a genome-wide analysis on the members of the maize ADF family. Thirteen ZmADF genes were compared with AtADFs and OsADFs and their phylogenetic classification was analyzed. In order to investigate ZmADFs specific functions in maize, we analyzed its expression pattern in different tissues in response to five abiotic stresses.

## 2. Results

### 2.1. Identification of ADF Genes in Maize

To identify the ADF gene family members in the maize genome, the maize reference genome was searched with BLASTP using InterPro domain (accession number IPR002108) as the query sequence. SMART (http.//smart.embl-heidelberg.de/) software was adopted to determine whether the ADF-H domain existed in the candidate ADF genes. Finally, 13 ADF-H domain-containing genes (*ZmADF1*-*ZmADF13*) were identified in this study, the number of ADF genes identified in maize (13) was similar to *Arabidopsis* (11) and rice (11). We also analyzed the gene location, open reading frame (ORF) length, amino acids length, molecular weight, iso-electric point (PI), and grand average of hydropathy (GRAVY) (Table 1). The length of the ORF varied from 369 to 576 bp. The isoelectric point of ZmADF genes ranged from 5.77 to 12.55, suggesting that the maize ZmADF proteins tend to alkaline. The molecular weight varied from 14.38 to 20.04 kDa and all studied ZmADF genes were below zero, which indicated that the 13 ZmADF proteins were all hydrophilic.

### 2.2. Chromosome Location and Gene Duplication of ADF Genes in Maize

**Figure 1:**
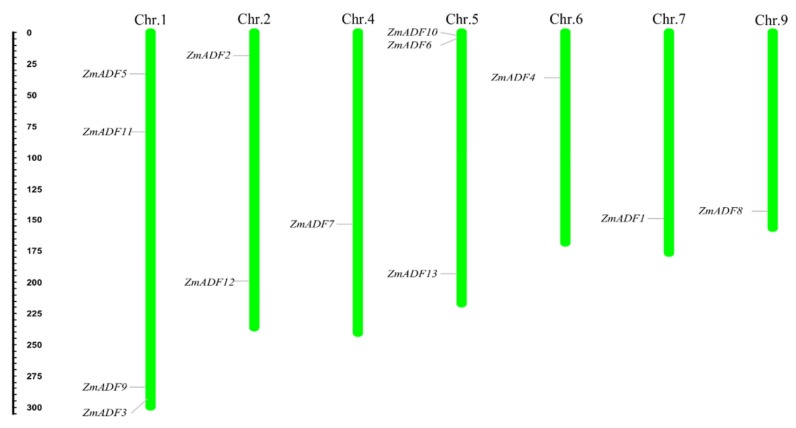
Chromosomal localization of maize ADFs. Thirteen ZmADF genes were distributed on chromosomes 1, 2, 4, 5, 6, 7, and 9. The chromosome numbers are indicated at the top of each vertical bar.

To show the location of 13 ZmADFs in the maize genome, we manually mapped the 13 ZmADFs on seven maize chromosomes in view of the MapChart results (Figure 1). The results show that 13 ZmADF genes distributed disparity, four ZmADFs were located on chromosome 1, including *ZmADF3*, *ZmADF5*, *ZmADF9,* and *ZmADF11*, three genes (*ZmADF6*, *ZmADF10,* and *ZmADF13*) on chromosome 5, two genes (*ZmADF2* and *ZmADF12*) on chromosome 2, and chromosomes 4, 6, 7 and 9 have one ADF gene, separately.

**Figure 2:**
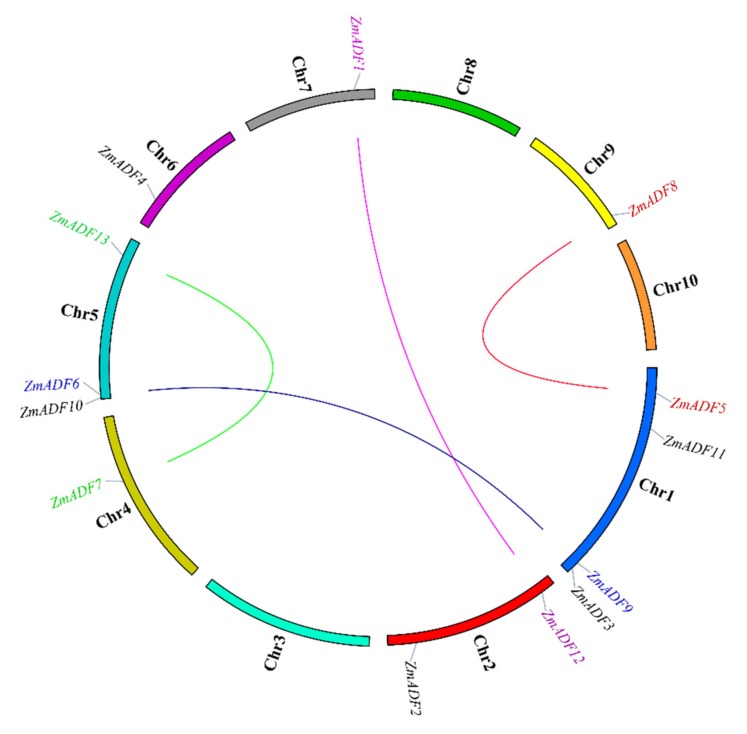
Gene duplication events of ZmADFs. The segmentally duplicated genes are linked by different color lines.

Segmental duplication is the most import driving force for generating a gene family during the evolution process. In the present study, based on sequences identity and query coverage, *ZmADF1/ZmADF12*, *ZmADF5/ZmADF8*, *ZmADF6/ZmADF9,* and *ZmADF7/ZmADF13* gene pairs were found to be segmentally duplicated (Figure 2), and were located on chromosomes 1, 2, 4, 5, 7, and 9. In contrast, none of the ADF genes in this study had tandem duplication.

### 2.3. Gene Structure of Identified ADF Genes in Maize

**Figure 3:**
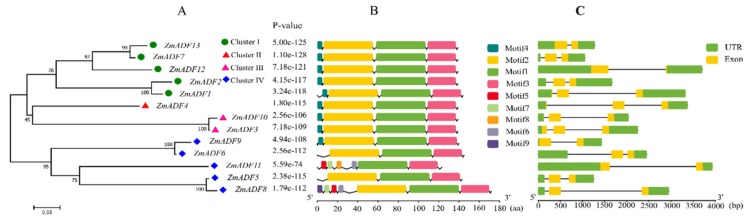
Conserved motifs and gene structure of the ZmADFs according to phylogenetic relationships. (**A**) The rootless neighbor-joining tree was constructed using MEGA7.0 program with the complete amino acid sequences of the 13 maize ADF proteins; (**B**) Conserved motif distribution map of the ZmADF gene. The 9 predicted motifs are represented by different colored boxes; **(C)** Exon and intron structure analyses of ZmADF genes were performed by TBtools. The green boxes, yellow boxes, and the black lines indicate UTRs region, exons, and introns, respectively. The length of the amino acid and exon and intron can be inferred by the ruler at the bottom.

The gene structure of ZmADF genes was analyzed by aligning the CDS region and the genomic sequence from the maize reference genome database (www.maizegdb.org) for each ZmADF gene (Figure 3C). Four ZmADF genes have two exons, and the remaining genes have three exons, indicating that all of these ZmADF genes’ structure are relatively simple. To further study the diversity of the ZmADF gene family, we also analyzed the conserved motifs using MEME online software (Figure 3B). A total of nine motifs were identified in this study (Figure S1). Due to the classifications based on phylogenetic analysis, these 13 genes could be divided into four clusters (Figure 3A). Motif 1 and Motif 3 were found in all 13 ZmADF genes, representing ADF-H/gelsolin-like domain. Motif 4 consisted of “MANAAS” domain and was detected in cluster Ⅰ, Ⅱ and Ⅲ. Motif 2 was lost in *ZmADF11* specificity. Except for cluster Ⅳ, these results showed that ZmADFs in the same group always shared similar motifs, and cluster Ⅳ showed motifs divergently, which could contribute to the functional divergence of the maize ADF family. According to a previous report, codon evolution of ADF genes has been investigated in relation to their putative functions, and codon position 26 in maize has been found to be important in forming new binding interactions, or possibly serves as a new target for protein regulation [10]. We analyzed the amino acid 26 in all ZmADF proteins and found that position 26 of most ZmADFs in cluster Ⅰ was S-Ser, except for *ZmADF1* (L-Leu). *ZmADF4* from cluster Ⅱ at position 26 was T-Thr. *ZmADF3* and *ZmADF10* from cluster Ⅲ at position 26 were L- Leu. Cluster Ⅳ which included *ZmADF9* (K-Lys), *ZmADF6* (E-Glu), *ZmADF11* (D-Asp), *ZmADF3* (K-Lys), and *ZmADF8* (Q-Gln) showed various amino acid sequences at position 26. Moreover, cluster Ⅳ also showed motifs divergently, indicating that amino acid 26 and motifs were related to the putative functions of ZmADFs.

### 2.4. Phylogenetic Classification of ADF Genes in Monocots, Dicots, and Mosses

**Figure 4:**
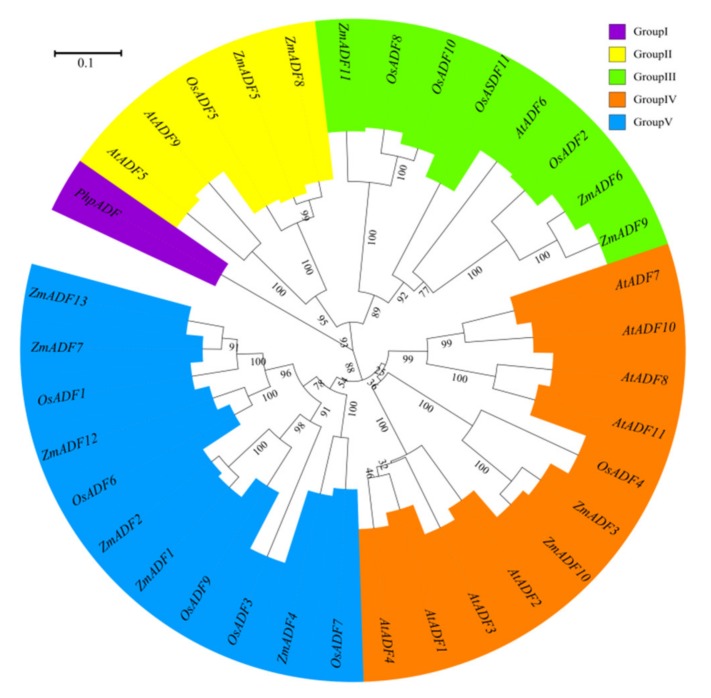
A phylogenetic tree of *Arabidopsis*, rice, and maize ADF proteins. The maximum likelihood (ML) method using MEGA7.0 software with 1,000 bootstrap replicates was adopted to construct the phylogenetic tree. Different types are marked with different colors.

To better understand the phylogenetic relationship among ADFs from common ancestry, ADF gene members from *Physcomitrella patens*, *Arabidopsis thaliana, Oryza sativa japonica,* and *Zea mays* were used to assess the evolutionary history of these protein families (see Table S1 for divergence times). MEGA7.0 was adopted to align the protein sequences and construct an unrooted phylogenetic tree by maximum likelihood method (Figure 4).

In addition to the PhpADF (group Ⅰ), the phylogenetic tree classified the ADF proteins into four main groups (group Ⅱ, Ⅲ, Ⅳ, V), consistent with published data of rice, *Arabidopsis,* and tomato [7,11]. In the phylogeny (Figure 4), group V included the largest numbers of ZmADFs proteins (*ZmADF1/2/4/7/12/13*), and five OsADFs also belonged to this group. Group IV also contained 11 members, including eight AtADFs, two ZmADFs (*ZmADF3/10*), and one OsADFs, followed by group Ⅲ, which included three numbers of ZmADFs (*ZmADF6/9/11*). Group Ⅱ only contained five ADF members, including *ZmADF5* and *ZmADF8* in this clade.

### 2.5. Expression Profiles of ZmADFs in Different Tissues and Orthologous Genes in Oryza sativa

**Figure 5:**
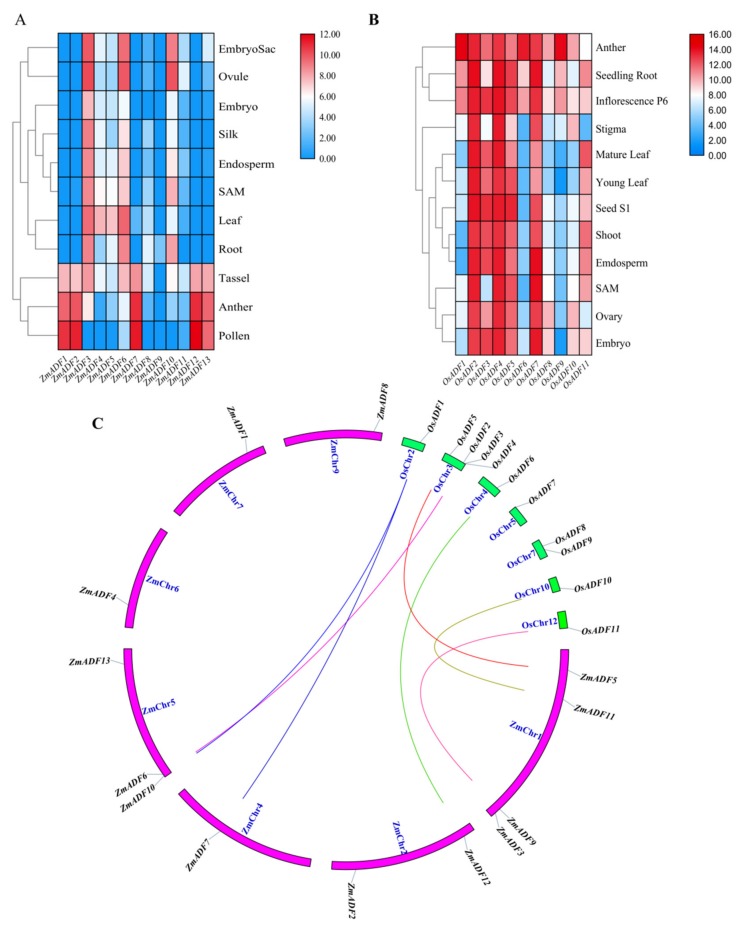
Expression profiles of ADF genes in maize and rice. (**A**) The expression of ADF gene family members in different tissues of maize; (**B**) Expression of OsADFs in different rice tissues; (**C**) Orthologous gene pairs between maize and rice. The letters SAM indicates stem apical meristem.

In an attempt to explore the possible functions of ZmADF genes in maize growth and development, the tissue expression profiles of ZmADF genes were determined in the embryo sac, ovule, embryo, silk, endosperm, SAM, leaf, root, tassel, anther, and pollen. These expression data showed that all ZmADF genes have different expression levels (Table S2) in 11 tissues at different developmental stages (Figure 5A). *ZmADF4*, *ZmADF5*, *ZmADF6*, *ZmADF10,* and *ZmADF3* showed relatively higher expression in all tissues, whereas *ZmADF9*, *ZmADF8* and *ZmADF11* showed very low levels of expression in most tissues and developmental stages. Interestingly, we found that *ZmADF1*, *ZmADF2*, *ZmADF7*, *ZmADF12,* and *ZmADF13* showed relatively high expression levels in tassel, anther, and pollen, which implied their crucial role in maize tassel development.

To validate ADF genes’ function in different species, we also retrieved the tissue expression profiles of OsADFs from Rice eFP Browser and analyzed their expression. As shown in Figure 5B, the expression level of OsADFs varied greatly in different tissues; OsADFs were highly expressed in anther, seedling root, and inflorescence P6, and lower expressed in SAM, ovary, and embryo. By combining the expression profiles between maize and rice, we identified that most ADFs showed very high expression levels in anther, pollen, and inflorescence, and showed very lower expression levels in SAM, endosperm, etc. (Figure 5A,B).

In this study, we also identified orthologous genes between maize and rice (Figure 5C) and aimed to validate whether orthologous genes had the same expression trends in different species. As shown in Figure 5, we concluded that orthologous gene pairs *OsADF1*/*ZmADF7*/*ZmADF10* and *OsADF6*/*ZmADF12* showed high expression levels in anther; *OsADF2*/*ZmADF6* and *OsADF11*/*ZmADF9* were highly expressed in root; *OsADF5*/*ZmADF5* showed the same expression pattern, with medium expression levels in leaf; and *OsADF10*/*ZmADF11* showed relatively higher expression in ovule.

### 2.6. Expression Patterns of ZmADFs under Different Abiotic Stresses

**Figure 6:**
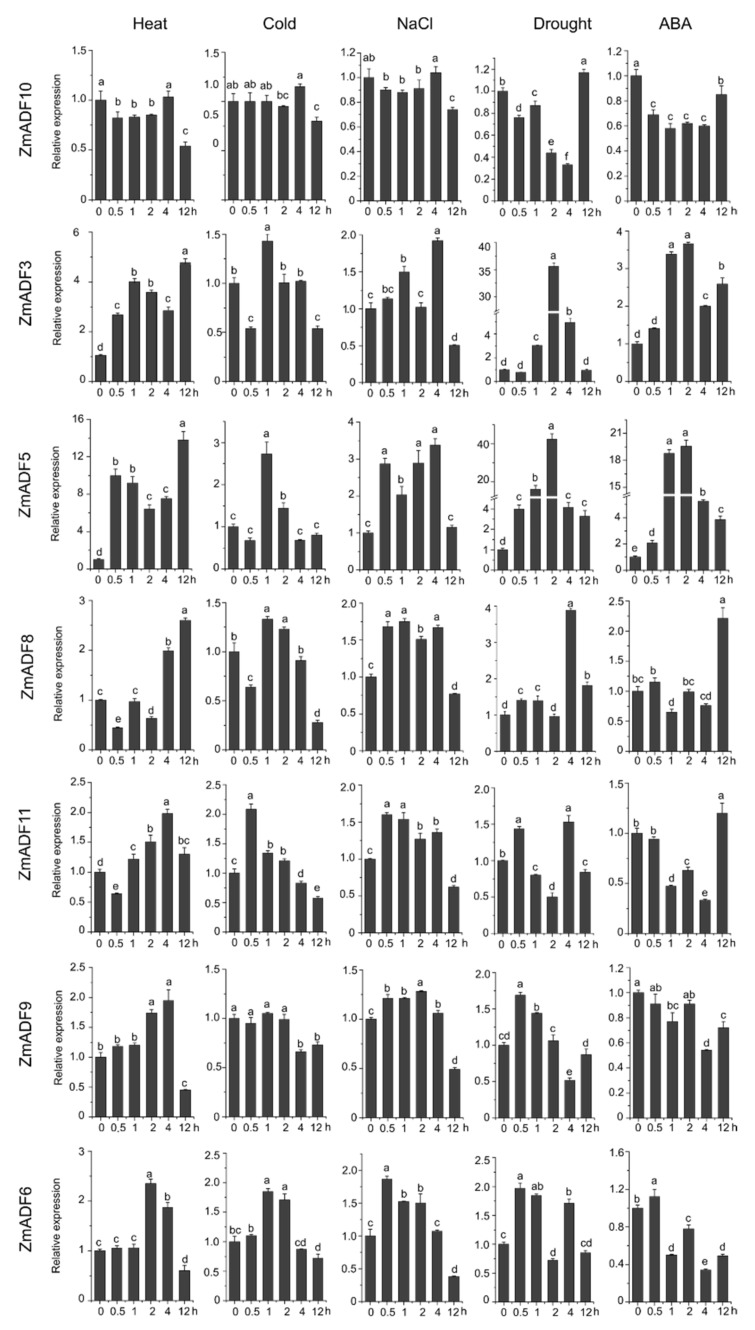

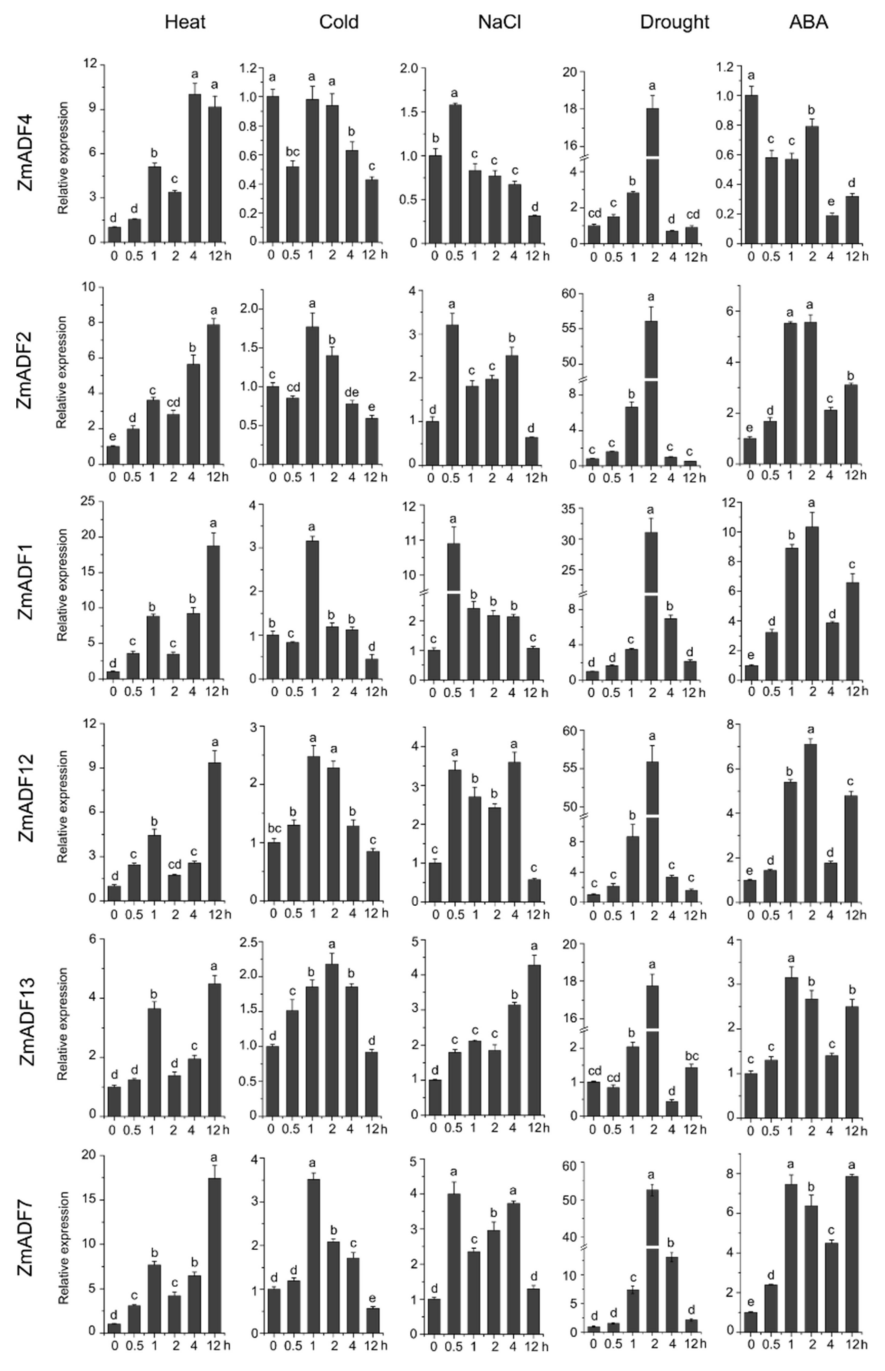
Relative expression levels of ZmADFs under heat (40 °C), cold (4 °C), salinity (0.2 M NaCl solution), drought, and abscisic acid (0.1 mM ABA) treatments in maize. qRT-PCR was used to study the expression level of 13 ZmADF genes. The internal reference gene is *ZmActin*. Three independent experiments were conducted. The abscissa represents the time point after stress treatment. Vertical bars indicate the standard error of mean. Small letter(s) above the bars indicated significant differences (*p* < 0.05, Duncan) between time stages.

In order to gain insights into the putative functions of ZmADF genes under abiotic stresses, we investigated their expression patterns in five abiotic stresses at different points (Figure 6). The qRT-PCR results revealed that five of the maize ADF genes (*ZmADF1*, *ZmADF7*, *ZmADF12*, and *ZmADF13* from group V and *ZmADF5* from group Ⅱ clade) were responsive to all abiotic stress treatments in this study. Moreover, *ZmADF1* was significantly upregulated in all abiotic stresses, indicating that ZmADF1 plays an important role in various abiotic stresses. *ZmADF2* and *ZmADF3* were significantly induced by heat, drought, and ABA treatments. *ZmADF2* could also be induced by NaCl. *ZmADF4* was upregulated under drought and heat treatments. *ZmADF6* was found only to be slightly upregulated under heat treatment.

For heat-treated samples, the expression levels of *ZmADF1/2/3/4/5/7/8/12/13* remained increased after 0.5 h of the heat treatment as compared with the control. However, *ZmADF6/9/11* were slightly induced until 4 h, and then decreased. *ZmADF10* was insensitive to the heat treatment. For cold-treated samples, first, *ZmADF11* was induced by cold treatment at 0.5 h, but then decreased. The expression levels of *ZmADF1/2/5/7/12* reached a peak at 1 h, and then decreased. Other ZmADFs were insensitive to the cold treatment. For NaCl-treated samples, the expression levels of *ZmADF1/2/5/7/13* were increased at 0.5 h, and then decreased, except for *ZmADF13* which continuously increased after 0.5 h and until 12 h of the NaCl treatment as compared with the control. Under drought and ABA treatments, the majority of the ZmADFs had similar expression characteristics. The expression levels of *ZmADF1/2/3/5/7/8/12/13* were significantly upregulated. Only *ZmADF4* exhibited the opposite expression pattern under drought and ABA treatments, which was downregulated in ABA and upregulated in drought treatment.

## 3. Discussion

### 3.1. Classification and Phylogenetic Analysis of the ADF Proteins among Monocots, Dicots and Mosses Indicate their Structural and Functional Similarities

The ADF protein family in plants is highly conserved both in structure and function. Our evolutionary history analysis of these protein families (Table S1) suggests that the origin and initial expansion of the ADF family (250 million years ago) predates the divergence of monocots and dicots 160 to 240 million years ago [35], indicating the functional importance of ADF genes in flowering plants. The phylogenetic classification of gene family members are important references for functional prediction. In this study, the phylogenetic classification of 36 ADF proteins from *Physcomitrella patens*, *Arabidopsis thaliana*, *Oryza sativa japonica*, and *Zea mays* were clustered into five groups. In addition to PhpADF (group Ⅰ), the ADF proteins from *Arabidopsis thaliana*, *Oryza sativa japonica,* and *Zea mays* were classified into four main groups (group Ⅱ, Ⅲ, Ⅳ, V), consistent with published data of rice, *Arabidopsis*, and tomato [7,11]. Codon evolution of ADF genes has been investigated in relation to their putative functions. Moreover, amino acid sequence at position 26 of ADF proteins in maize was found to be important in forming new binding interactions, or possibly serves as a new target for protein regulation [10]. In this study, we found that position 26 of most ZmADFs in group V was Ser, which was consistent with the members which were specifically expressed in pollen and significantly responded to drought and heat stress. *ZmADF3* and *ZmADF10* from group Ⅳ at position 26 were L-Leu and they expressed higher in all tissues, suggesting that amino acid 26 was related to the putative functions of ZmADFs. In addition, OsADFs and ZmADFs, in the same group, possess a similar function. For instance, *OsADF1/6/9* and *ZmADF1/2/7/12/13* from group V predominately expressed in anther and pollen. *OsADF3* was highly induced under drought stress in rice shoot (Figure S2), in the same group, *ZmADF1/2/4/7/12/13* were also drastically induced under drought stress (Figure 6).

Following the whole genome duplication events, the genes from a common ancestor that formed a gene family in regions of some duplicated genes could become pseudogenes while the remaining genes were retained and either preserved or evolved with various functions [9]. Four pairs of segmental duplicated genes of ZmADF genes were detected (Figure 2). Among these, *ZmADF5*/*ZmADF8* and *ZmADF6*/*ZmADF9* are from group Ⅱ and group Ⅲ separately, and the expression levels of *ZmADF5* and *ZmADF6* were high in all detected tissues, but that of *ZmADF8* and *ZmADF9* were lower in all detected tissues and in the five abiotic stresses, suggesting the *ZmADF8* and *ZmADF9* were pseudogenes. On the other hand, *ZmADF1*/*ZmADF12* and *ZmADF7*/*ZmADF13* from group V were all specifically expressed in anther and pollen, and they were significantly upregulated in drought stress, indicating that duplicated genes can strengthen the function of ancestral genes.

The orthologous gene pairs between rice and maize from the same group also showed a similar expression pattern. For example, *OsADF1*/*ZmADF7* and *OsADF6*/*ZmADF12* showed high expression levels in anther; *OsADF2/ZmADF6* and *OsADF11*/*ZmADF9* were highly expressed in root; *OsADF5*/*ZmADF5* showed the same expression pattern, with medium expression levels in leaf; and *OsADF10*/*ZmADF11* showed relatively higher expression in ovule, also indicating that the members of gene family from the same group could have a similar function.

### 3.2. To Protect Or Promote the Reproductive Development, which is more Important?

Currently, maize has become one of the most important crops in the world. Unfortunately, during the period of growth and development, maize suffers from numerous stresses. Nowadays, thanks to the publication of the maize genome sequence and the development of bioinformatics, the identification and analysis of gene families has become less difficult. Summarizing the important gene families related to plant growth, development, and stress resistance helps to study the specific function of genes, and therefore provide a theoretical basis for genetic improvement. It can also provide a valuable reference for the study of candidate genes obtained by map-based cloning. The ADF gene family has been reported to play a crucial role in growth, development, and response to abiotic stresses. However, the genome-wide identification of ADF gene family in maize has not been reported. In our study, 13 ZmADF genes were identified from maize (B73) genome, and several analyses of bioinformatics and expression profiles were conducted. Consistent with the reports of the ADF family classification in other plants [7,11], ADF genes in maize were also classified into four groups. The members belonging to the same groups usually had similar expression profiles in tissues and stresses, indicating they could drive analogous functions. Moreover, according to previous reports, each family can be subdivided into two classes that differ in their expression patterns, i.e., reproductive or constitutive and vegetative [34,36,37,38]. In our study, *ZmADF1/2/7/12/13* from group V expressed specifically in tassel, anther, and pollen, other ZmADF genes expressed in all tissues or vegetative tissues. Pollen and germinating pollen specific expression of *ZmADF1* and *ZmADF2* (previously named maize actin-binding protein, ZmABP1 and ZmABP2, in [13,34]) suggest that the protein products can be involved in pollen actin reorganization. *ZmADF3* (previously named ZmABP3) was expressed in all vegetative tissues examined, whereas it was suppressed in pollen and germinated pollen [13]. However, there is currently no report on whether pollen dysplasia or pollen sterility occurs after loss of the gene function of *ZmADF1* and *ZmADF2*. Mutants have only been reported on *ZmADF3*, demonstrating that *ZmADF3* binding of F-actin could be spatially distinguished from that of G-actin [33]. In the present research, the expression of *ZmADF1* and *ZmADF2* in maize two-week-old seedlings is induced by several stresses, especially under drought treated stress, indicating that the pollen-specific expressed *ZmADF1* and *ZmADF2* not only play an important role in pollen actin reorganization but also function to resist external environmental stresses. It is not known whether their function is more important in reproductive development or in stress response. For example, RNAi and downregulation mutant lines of a meiosis-enriched gene *ZmAGO18b*, which is specifically expressed in tapetum cells, pollen mother cells (PMCs), and microspores of meiotic tassel [39], did not show morphological abnormalities and sterility in tassel development. However, the reduced expression of *ZmAGO18b* in those plant lines resulted in increased height as compared with the control lines [40]. Furthermore, *ZmAGO18b* could be significantly induced in seedlings by drought treatment [41]. Therefore, we speculate that the genes specifically expressed in the reproductive organs, in addition to playing a possible role in reproductive development, could have a more important function to protect the reproductive development, by braking in time to cope with the harsh external environment that can be suffered during the development stage. This shows that in the process of evolution, the organism has developed the reproductive stage into the most important period and avoided external interference to ensure normal reproductive development. These reproductive-specific genes could be used as important candidate genes for stress resistance research and plant resistance breeding.

## 4. Materials and Methods

### 4.1. Identification and Analysis of ADF Family Genes

The whole protein sequences, coding sequences (CDS), mRNA sequences and genome sequences of maize (inbred line B73) were downloaded from MaizeGDB (https.//www.maizegdb.org/, version 2). In total, we identified 13 ADF genes in maize using the InterPro domain accession number IPR002108 (the ADF-H domain) and using BLAST searches of nucleotides in maize reference genome, all redundant protein sequences were discarded in this study. In order to confirm the putative ADF genes in the maize genome, Pfam and SMART (http.//smart.embl-heidelberg.de/, version 7.0) were used to confirm the presence of ADF domains. Protein isoelectric point (PI) and molecular weight (Mw) of each gene product were calculated using ExPasy and parameter was set to average [42].

### 4.2. Phylogenetic Analysis of Maize ADF Genes

In order to study the phylogenetic relationship, the ADF protein sequences of *Arabidopsis* and rice from the published article were download [11], and this accuracy was confirmed by searching the TAIR (https.//www.arabidopsis.org/) and RGAP (http.//rice.plantbiology.msu.edu/) databases with manual corrections. The non-vascular bryophyte Physcomitrella patens was used to root the tree. Multiple sequence alignment of maize ADF protein sequences and the orthologous genes from *Arabidopsis* and rice was performed with MUSCLE software (version 3.6) with default parameters [43]. The aligned sequences were subjected to phylogenetic analysis by the maximum likelihood method using MEGA (version 7.0) with 1,000 bootstrap replicates [44]. Online software iTOL (Interactive Tree of Life, version 5) was employed to draw and manage the phylogenetic tree [45]. 

### 4.3. Analysis of Chromosome Location and gene Duplication

The chromosomal locations of the 13 maize ADF genes were determined according to maize reference genome information [46]. The MapChart software (version 2.0) was used to map their positions along the 10 chromosomes [47]. Duplication analysis of all identified ZmADFs was investigated according to Guo’s method, which defined the gene duplication with the following 3 criteria: (1) the length of the aligned segments covered more than 80% of the longest gene, (2) the identity of aligned region was more than 80%, and (3) only one duplication event was identified for tightly linked genes [48]. The map was drafted using Tbtools software (https.//github.com/CJ-Chen/TBtools).

### 4.4. Gene Structure Analysis

Exon and intron organizations for all ZmADF genes were obtained by using the online Gene Structure Display Server (GSDS, version 2.0) [49] via aligning the cDNAs to the corresponding gene sequences. MEME (Multiple EM for Motif Elicitation, http.//meme-suite.org/tools/meme, version 5.1.1) was adopted to identify the possible conserved motifs in the complete amino acid sequences of maize ADF proteins setting a maximum number of motifs to 10. 

### 4.5. Expression Pattern Analysis in Different Tissues of ZmADF Genes and Orthologous Genes in Oryza sativa

To determine the expression patterns of the ADF genes in maize tissues, expression profiles based on microarray data were collected from the Maize eFP Browser (http.//bar.utoronto.ca/efp_maize/cgi-bin/efpWeb.cgi), which included embryo sac, ovule, embryo, silk, endosperm, SAM, leaf, root, tassel, anther, and pollen. These public data sources are all based on the experimental data from maize inbred line B73. Rice expression data were retrieved from the Rice eFP Blowser (http://bar.utoronto.ca/transcriptomics/efp_rice/cgi-bin/efpWeb.cgi), which included seedling root, anther, inflorescence, mature leaf, young leaf, stigma, seed, shoot, endosperm, SAM, ovary, and embryo. All expression values were normalized by log10 transformed and displayed using a heat map to analyze corresponding genes’ expression pattern in different tissues. The heat map was generated using Tbtools software (https.//github.com/CJ-Chen/TBtools). To profile orthologous gene pairs’ expression level between maize and rice, we retrieved the orthologous gene pairs from the Rice Genome Annotation Project (http://rice.plantbiology.msu.edu/index.shtml).

### 4.6. Plant Materials and qPCR Expression Analysis under Diverse Abiotic Stresses

The selected seeds of maize (*Zea mays* L. cvB73) were surface sterilized and repeatedly rinsed with tap water, the seeded in Hoagland nutrient solution after immersion and imbibition for 12 h, cultured in potting sand in the greenhouse conditions at 25 °C with a 16 h light and 8 h dark cycle (Department of Biochemistry and Molecular Biology of Hubei University of Arts and Science, Xiangyang, China). Two-week-old seedlings growing identically were processed with heat (40 °C), cold (4 °C), salinity (0.2 M NaCl solution), drought, and abscisic acid (0.1 mM ABA). The seedlings were subjected to abiotic stress treatments basically as described by Zhai et al. (2019) [41]. We selected three individual and whole seedlings as three independent biological replicates at each treatment point (0, 0.5, 1, 2, 4, and 12 h) separately. Extract total RNA was extracted by Trizol (Invitrogen, Carlsbad, CA, USA) and the method followed the protocol of instruction. M-MLV (Invitrogen) reverse transcriptase and an oligo (dT) primer were used to synthesize first-strand cDNA. qRT-PCR was performed on the ABI7500 using the SYBR FAST qPCR Kit Master Mix (2x) LightCycler (KAPA, Boston, MA, USA). The *ZmActin* gene was used as the internal control. The primer sequences of ZmADFs and *ZmActin* are in Table S3. The 2^−∆∆Ct^ method in Excel was used to calculate the relative gene expression [50].

## 5. Conclusions

The plant ADF family plays key roles in growth, development, and defense-related functions. However, there has been a lack of genome-wide information of this family in maize. In this study, a total of 13 ADF genes were first identified in maize. According to the alignment of the protein sequences, the ZmADF proteins were divided into four groups. Subsequently, we used a variety of bioinformatics methods to analyze chromosomal location, gene duplication, and gene structure. Moreover, the expression patterns of ZmADFs in different tissues and abiotic stresses were analyzed by microarray data and qRT-PCR, respectively. The results showed that ZmADF genes from group V (*ZmADF1*, *ZmADF2*, *ZmADF7,* and *ZmADF12*) had high expression levels in tassel, anther, and pollen, implying ZmADFs from group Ⅱ could play important roles in the reproductive development stage. However, the expression of *ZmADF1*, *ZmADF7*, *ZmADF12,* and *ZmADF13* from group V were also remarkably induced by multiple stresses in this study, suggesting that these genes have much more important tolerance functions in maize, especially in the reproductive stage. The results of our study build the foundation for functional research into the ZmADF genes and also provide a valuable reference for stress-resistant breeding of maize.

## Figures and Tables

**Table 1:** Detailed information of all actin-depolymerizing factor (ADF) family genes identified in the maize genome.

Gene Name	Location	Accession Number	Open Reading Frame Length (bp)	Amino Acids (aa)	Isoelectric Point	Molecular Weight (KD)(Kda)	GRAVY
*ZmADF1*	7:148879558..148880837(F)	GRMZM2G117603	432	144	6.32	16.54	−0.586
*ZmADF2*	2:199484159..199485220(R)	GRMZM2G097122	417	139	5.57	16.08	−0.642
*ZmADF3*	1:293162401..293166106(F)	GRMZM2G060702	414	138	12.55	15.63	−1.231
*ZmADF4*	6:35896131..35897803(R)	GRMZM2G037140	417	139	7.66	15.86	−0.369
*ZmADF5*	1:32867955..32871278(R)	GRMZM2G077942	429	143	8.41	16.41	−0.287
*ZmADF6*	5:5245812..5249187(F)	GRMZM2G130678	435	145	6.15	16.83	−0.471
*ZmADF7*	4:153268732..153270773(R)	GRMZM2G463471	417	139	6.31	15.86	−0.316
*ZmADF8*	9:143174940..143177192(F)	GRMZM2G147775	516	172	9.51	20.04	−0.505
*ZmADF9*	1:284216333..284217771(F)	GRMZM2G108807	420	140	7.78	16.41	−0.469
*ZmADF10*	5:2362323..2364777(R)	GRMZM2G002825	417	139	5.47	15.91	−0.485
*ZmADF11*	1:80054390..80058323(R)	GRMZM2G064875	369	123	5.6	14.38	−0.667
*ZmADF12*	2:18815692..18816950(F)	GRMZM2G071327	417	139	5.27	15.98	−0.570
*ZmADF13*	5:193084737..193087687(R)	GRMZM2G015127	417	139	7.56	15.89	−0.271

## Supplementary Materials

Supplementary materials can be found at https://www.mdpi.com/1422-0067/21/5/1751/s1.

## Abbreviations

ADF	Actin-depolymerizing factor
SAM	Shoot apical meristem
GPA	Green peach aphid
ABP	Actin-binding protein
ORF	Open reading frame
CDS	Coding sequences
ROS	Reactive oxygen species
GRAVY	Grand average of hydropathy
PI	Iso-electric point
Mw	Molecular weight
HMM	Hidden Markov Model
PMCs	Pollen mother cells; 2,4-D: 2,4-dichlorophenoxyacetic acid
ABA	Abscisic acid
GA3	Gibberellic acid
IAA	Indole-3-acetic acid
JA	Jasmonic acid
HR	Hypersensitive response
qRT-PCR	Quantitative real-time PCR
