# Supplementary material for: Genome-Wide Identification and Characterization of Actin-Depolymerizing Factor (ADF) Family Genes and Expression Analysis of Responses to Various Stresses in Zea Mays L
Source: Int J Mol Sci. 2020 Mar 4;21(5):1751. doi: 10.3390/ijms21051751 (PMC7084653; doi:10.3390/ijms21051751)
Supplement: Supplementary file 1 [file ijms-21-01751-s001.zip › Figure Legend.docx]

**Figure 1. Chromosomal localization of maize ADFs.** Thirteen ZmADF genes were distributed on chromosomes 1, 2, 4, 5, 6, 7 and 9. The chromosome numbers are indicated at the top of each vertical bar.

**Figure 2. Gene duplication events of** **ZmADFs.** The segmentally duplicated genes are linked by different color lines.

**Figure 3. Conserved motifs and gene structure of the ZmADFs according to phylogenetic relationships.** The rootless Neighbor-Joining tree was constructed using MEGA7.0 program with the complete amino acid sequences of the 13 maize ADF proteins. Conserved motif distribution map of the ZmADF gene. The 9 predicted motifs are represented by different colored boxes. Exon-intron structure analyses of ZmADF genes were performed by TBtools. The green boxes, yellow boxes, and the black lines indicate UTRs region, exons, and introns, respectively. The length of the amino acid and exon-intron can be inferred by the ruler at the bottom.

**Figure 4. A phylogenetic tree of Arabidopsis, rice and maize ADF proteins.** The maximum likelihood (ML) method using MEGA7 software with 1000 bootstrap replicates was adopted to construct the phylogenetic tree. Different types are marked with different colors.

**Figure 5. Expression profiles of ADF genes in maize and rice.** (A) The expression of ADF gene family members in different tissues of maize; (B) Expression of OsADFs in different rice tissues; (C) Orthologous gene pairs between maize and rice. The letters SAM indicates stem apical meristem.

**Figure 6. Relative expression level of ZmADFs under heat (40˚C), cold (4˚C), salinity (0.2 M NaCl solution), drought and abscisic acid (0.1 mM ABA) treatments in maize.** qRT-PCR was used to study the expression level of 13 ZmADF genes. The internal reference gene is ZmActin. Three independent experiments were conducted. The abscissa represents the time point after stress treatment. Vertical bars indicate the standard error of mean. Small letter(s) above the bars indicated significant differences (P<0.05, Duncan) between time stages.

**Table1. Detailed information of all ADF family genes identified in the maize genome.**

**Figure S1. The amino acid sequences of 9 conserved motifs.**

**Figure S2. Relative expression level of OsADFs under drought, salt and cold treatments in rice.**

**Table S1. Plant species sampled for ADF sequences and their divergence times from a common ancestor with Arabidopsis.**

**Table S2. The expression data of ZmADFs in 11 different tissue from webset.**

**Table S3. The qRT-PCR primer sequences of ZmADF genes and ZmActin gene.**
