# Supplementary material for: Genome-Wide Identification and Characterization of Actin-Depolymerizing Factor (ADF) Family Genes and Expression Analysis of Responses to Various Stresses in Zea Mays L
Source: Int J Mol Sci. 2020 Mar 4;21(5):1751. doi: 10.3390/ijms21051751 (PMC7084653; doi:10.3390/ijms21051751)
Supplement: Supplementary file 1 [file ijms-21-01751-s001.zip › Supplementary Table S1.docx]

**Supplementary Table 1 Plant species sampled for ADF sequences and their divergence times from a common ancestor with Arabidopsis**

| **Species** | **Informative Classification** | **Estimated Divergence Times** | **Ascension #** |
| --- | --- | --- | --- |
| *Physcomitrella patens* | Non-vascular plant, Bryophyta | 600 mya | XP_001777224 |
| *Zea mays* | Angiosperm, monocot, Poaceae | 250 mya | GRMZM2G117603  GRMZM2G097122  GRMZM2G060702  GRMZM2G037140  GRMZM2G077942  GRMZM2G130678  GRMZM2G463471  GRMZM2G147775  GRMZM2G108807  GRMZM2G002825  GRMZM2G064875  GRMZM2G071327  GRMZM2G015127 |
| *Oryza sativa japonica* (rice) | Angiosperm, monocot, Poaceae | 250 mya | LOC_Os02g44470  LOC_Os03g56790  LOC_Os03g60580  LOC_Os03g60590  LOC_Os03g13950  LOC_Os04g46910  LOC_Os05g02250  LOC_Os07g20170  LOC_Os07g30090  LOC_Os10g37670  LOC_Os12g43340 |
| *Arabidopsis thaliana* | Angiosperm, dicot, Brassicaceae | 0 mya | AT3G46010  AT3G46000  AT5G59880  AT5G59890  AT2G16700  AT2G31200  AT4G25590  AT4G00680  AT4G34970  AT5G52360  AT1G01750 |
