# Supplementary material for: Genome-Wide Identification and Characterization of Actin-Depolymerizing Factor (ADF) Family Genes and Expression Analysis of Responses to Various Stresses in Zea Mays L
Source: Int J Mol Sci. 2020 Mar 4;21(5):1751. doi: 10.3390/ijms21051751 (PMC7084653; doi:10.3390/ijms21051751)
Supplement: Supplementary file 1 [file ijms-21-01751-s001.zip › Table 1.docx]

| **Gene Name** | **Location** | **Accession Number** | **Open Reading Frame Length (bp)** | **Amino Acids(aa)** | **Isoelectric Point** | **Molecular Weight (KD)(Kda)** | **GRAVY** |
| --- | --- | --- | --- | --- | --- | --- | --- |
| ZmADF1 | 7:148879558..148880837(F) | GRMZM2G117603 | 432 | 144 | 6.32 | 16.54 | -0.586 |
| ZmADF2 | 2:199484159..199485220(R) | GRMZM2G097122 | 417 | 139 | 5.57 | 16.08 | -0.642 |
| ZmADF3 | 1:293162401..293166106(F) | GRMZM2G060702 | 414 | 138 | 12.55 | 15.63 | -1.231 |
| ZmADF4 | 6:35896131..35897803(R) | GRMZM2G037140 | 417 | 139 | 7.66 | 15.86 | -0.369 |
| ZmADF5 | 1:32867955..32871278(R) | GRMZM2G077942 | 429 | 143 | 8.41 | 16.41 | -0.287 |
| ZmADF6 | 5:5245812..5249187(F) | GRMZM2G130678 | 435 | 145 | 6.15 | 16.83 | -0.471 |
| ZmADF7 | 4:153268732..153270773(R) | GRMZM2G463471 | 417 | 139 | 6.31 | 15.86 | -0.316 |
| ZmADF8 | 9:143174940..143177192(F) | GRMZM2G147775 | 516 | 172 | 9.51 | 20.04 | -0.505 |
| ZmADF9 | 1:284216333..284217771(F) | GRMZM2G108807 | 420 | 140 | 7.78 | 16.41 | -0.469 |
| ZmADF10 | 5:2362323..2364777(R) | GRMZM2G002825 | 417 | 139 | 5.47 | 15.91 | -0.485 |
| ZmADF11 | 1:80054390..80058323(R) | GRMZM2G064875 | 369 | 123 | 5.6 | 14.38 | -0.667 |
| ZmADF12 | 2:18815692..18816950(F) | GRMZM2G071327 | 417 | 139 | 5.27 | 15.98 | -0.570 |
| ZmADF13 | 5:193084737..193087687(R) | GRMZM2G015127 | 417 | 139 | 7.56 | 15.89 | -0.271 |

Table 1 Detailed information of all ADF family genes identified in the maize genome
