# Supplementary figures and images for: Genome-Wide Identification and Characterization of Actin-Depolymerizing Factor (ADF) Family Genes and Expression Analysis of Responses to Various Stresses in Zea Mays L
Source: Int J Mol Sci. 2020 Mar 4;21(5):1751. doi: 10.3390/ijms21051751 (PMC7084653; doi:10.3390/ijms21051751)

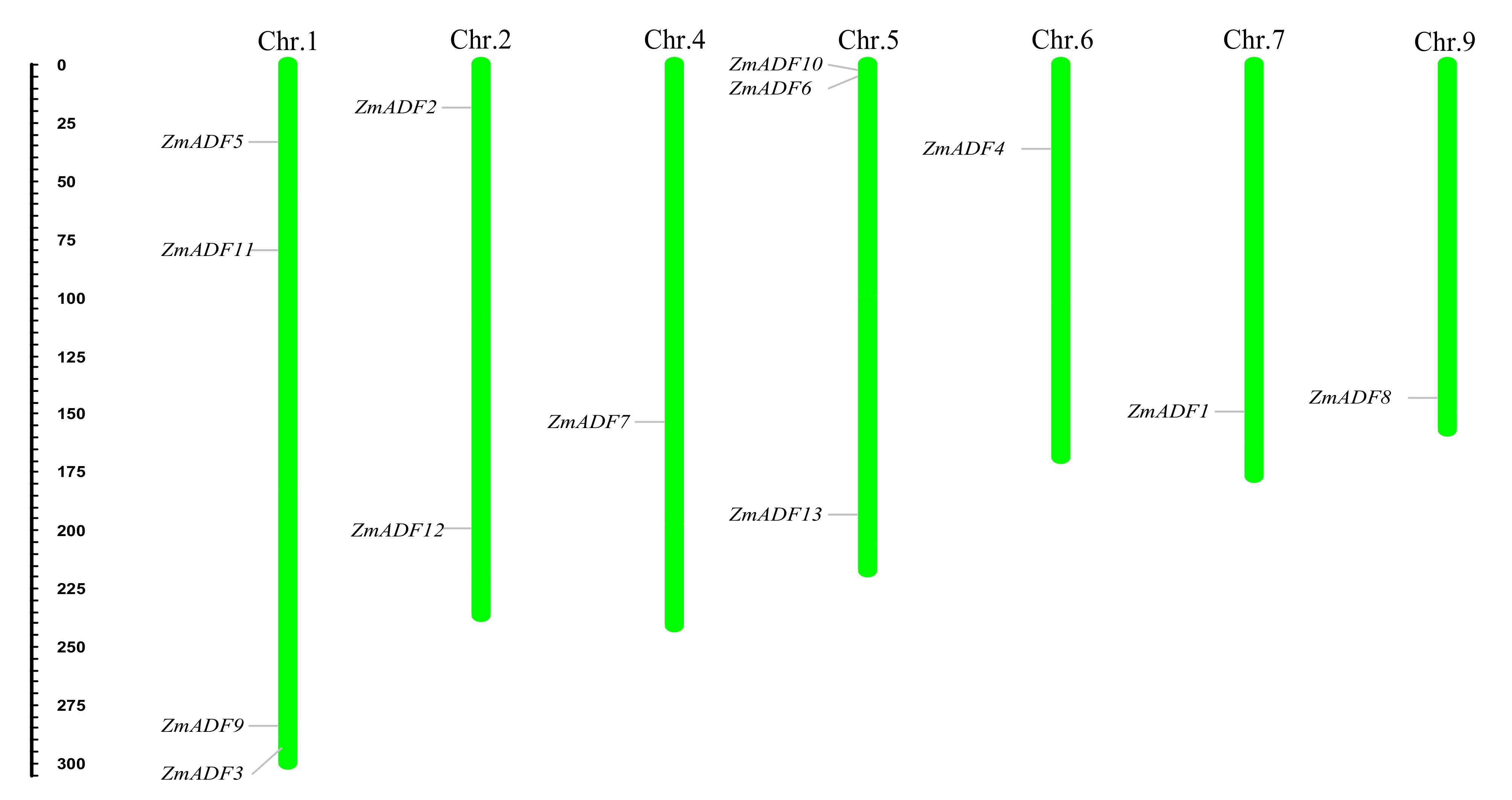

Supplement: Supplementary file 1 [file ijms-21-01751-s001.zip › Figure 1.tiff]

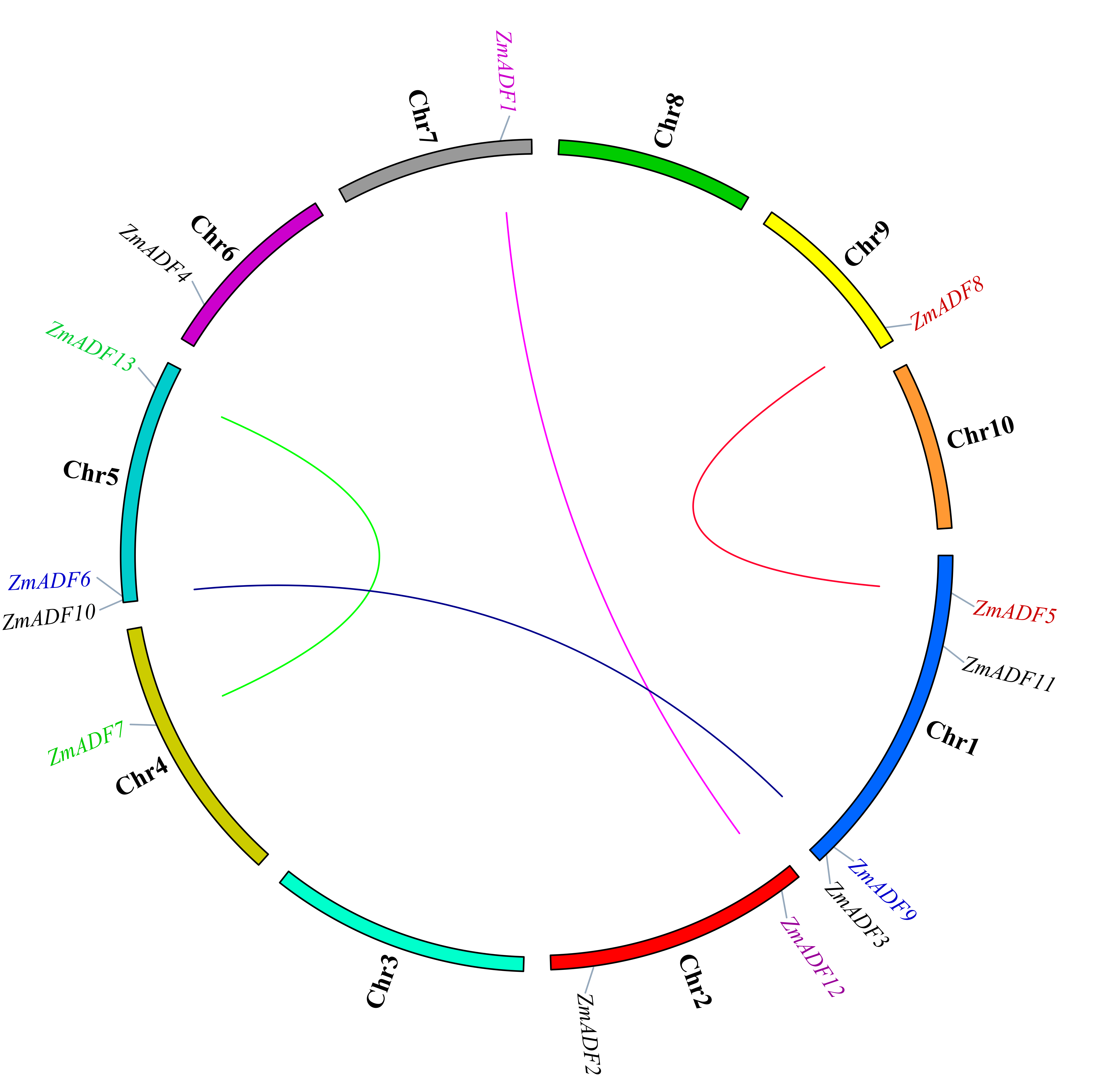

Supplement: Supplementary file 1 [file ijms-21-01751-s001.zip › Figure 2.tiff]

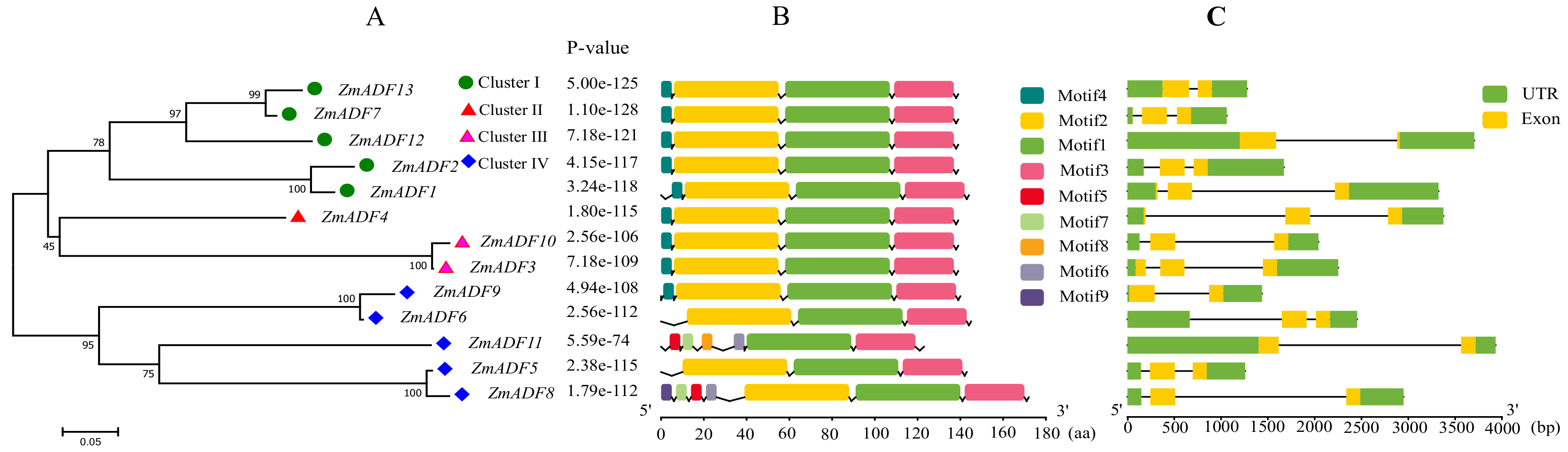

Supplement: Supplementary file 1 [file ijms-21-01751-s001.zip › Figure 3.tiff]

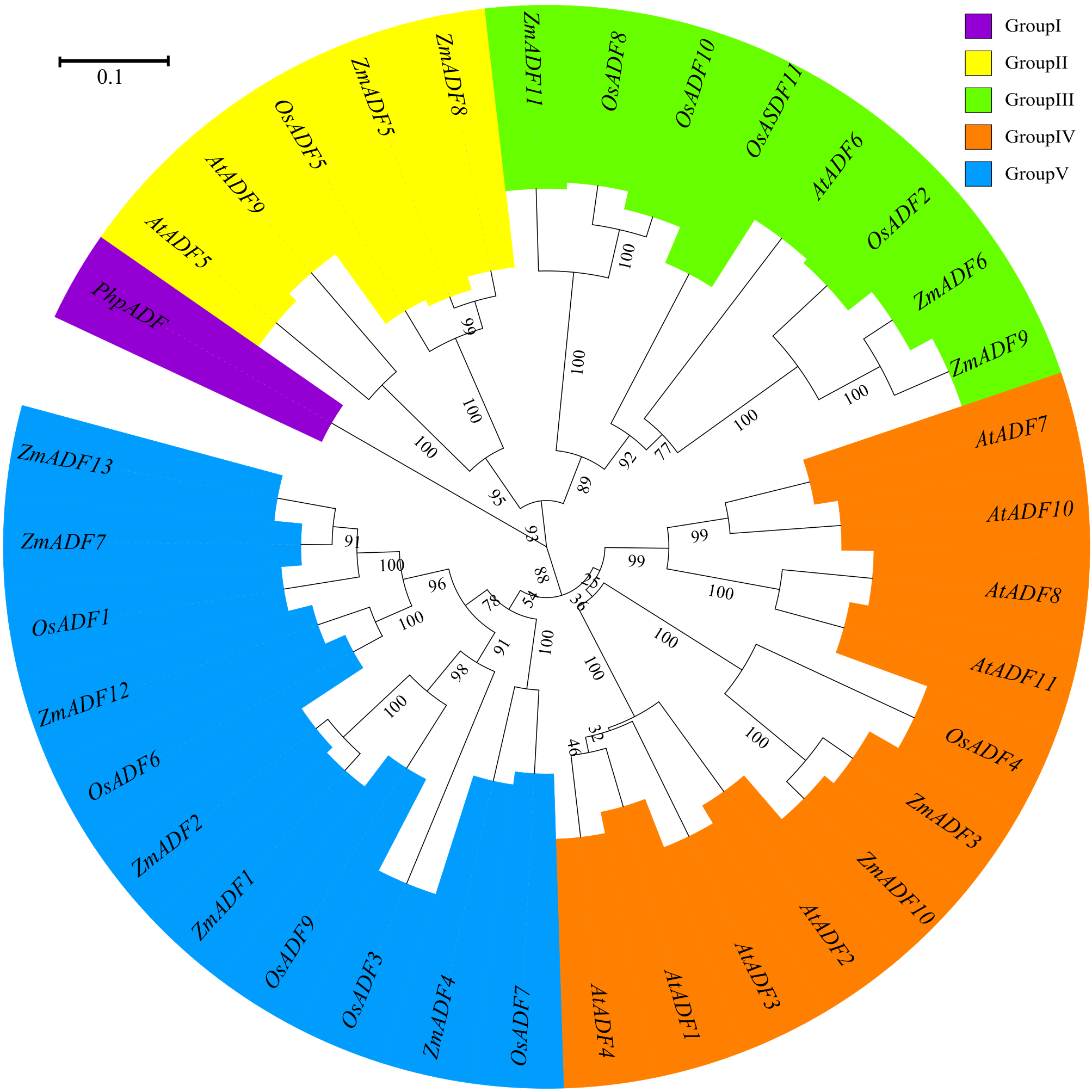

Supplement: Supplementary file 1 [file ijms-21-01751-s001.zip › Figure 4.tiff]

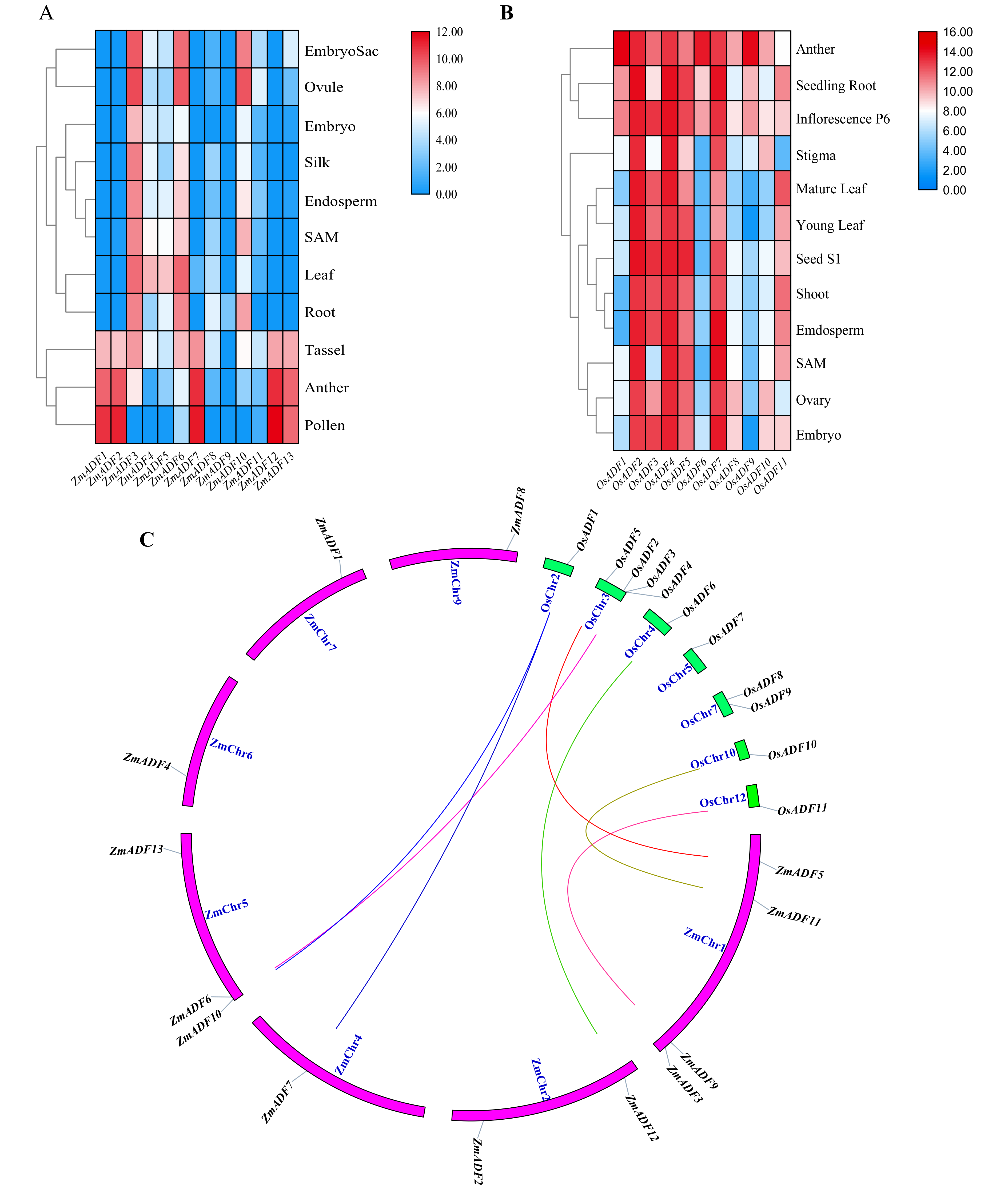

Supplement: Supplementary file 1 [file ijms-21-01751-s001.zip › Figure 5.tiff]

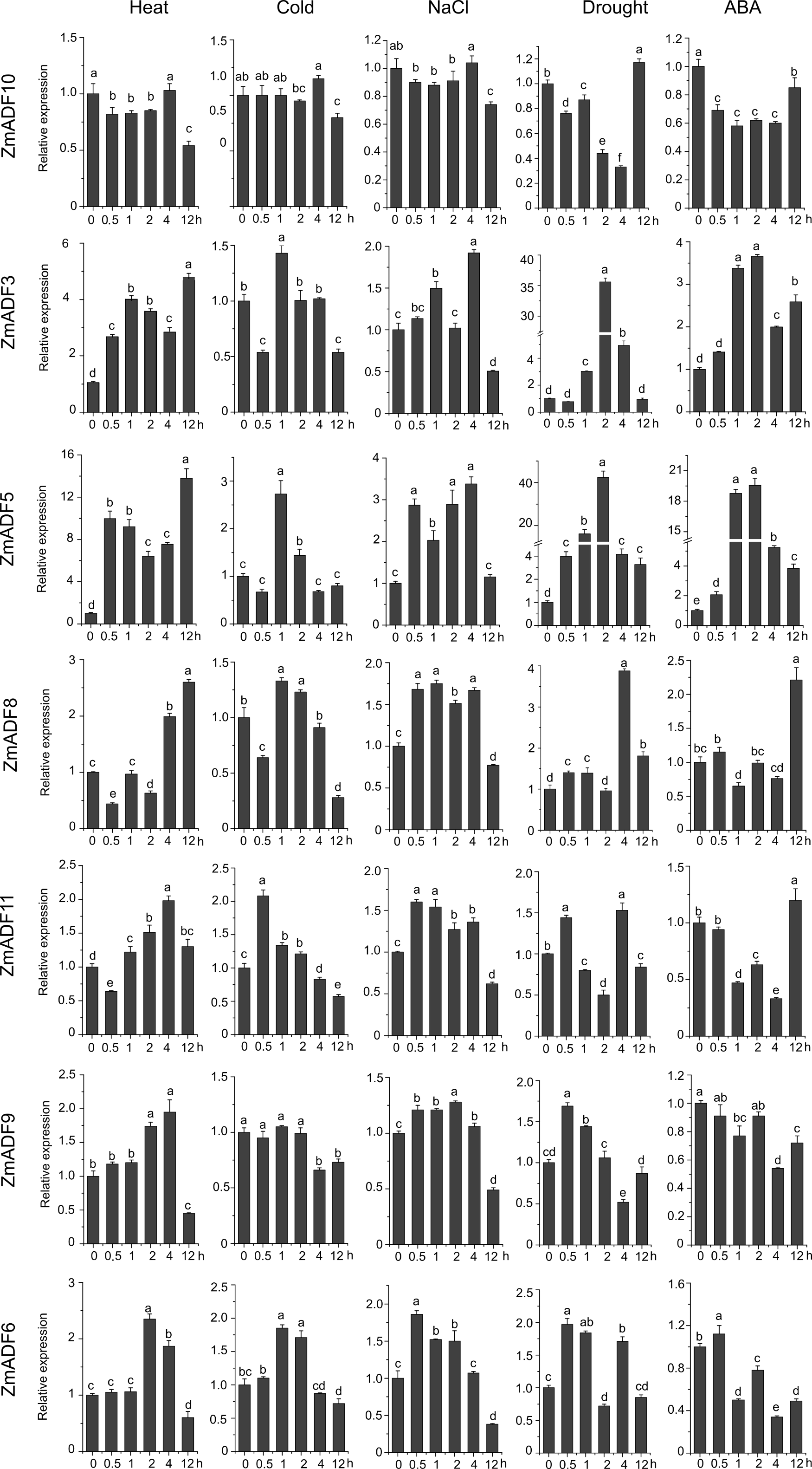

Supplement: Supplementary file 1 [file ijms-21-01751-s001.zip › Figure 6-1.tiff]

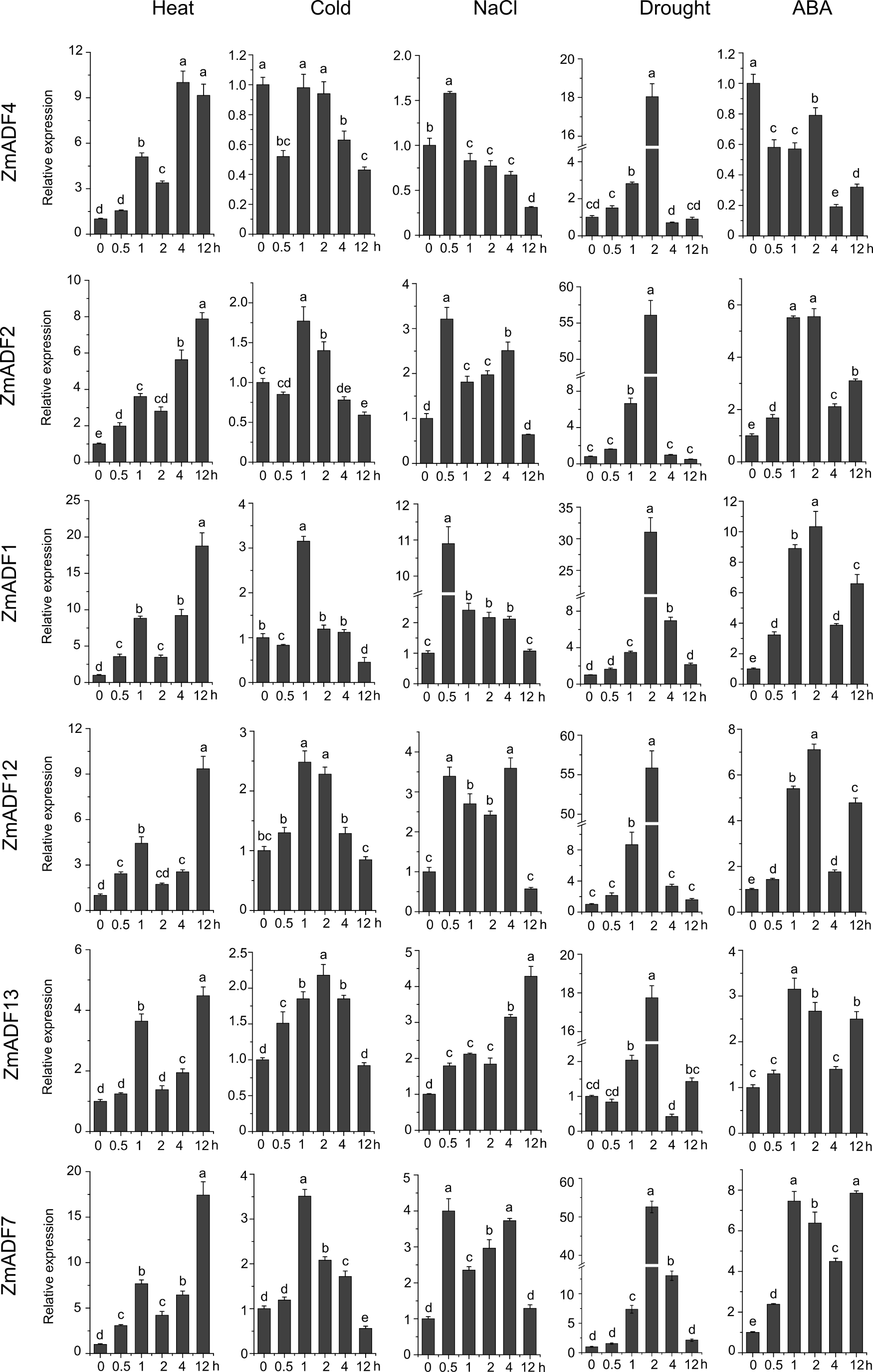

Supplement: Supplementary file 1 [file ijms-21-01751-s001.zip › Figure 6-2.tiff]

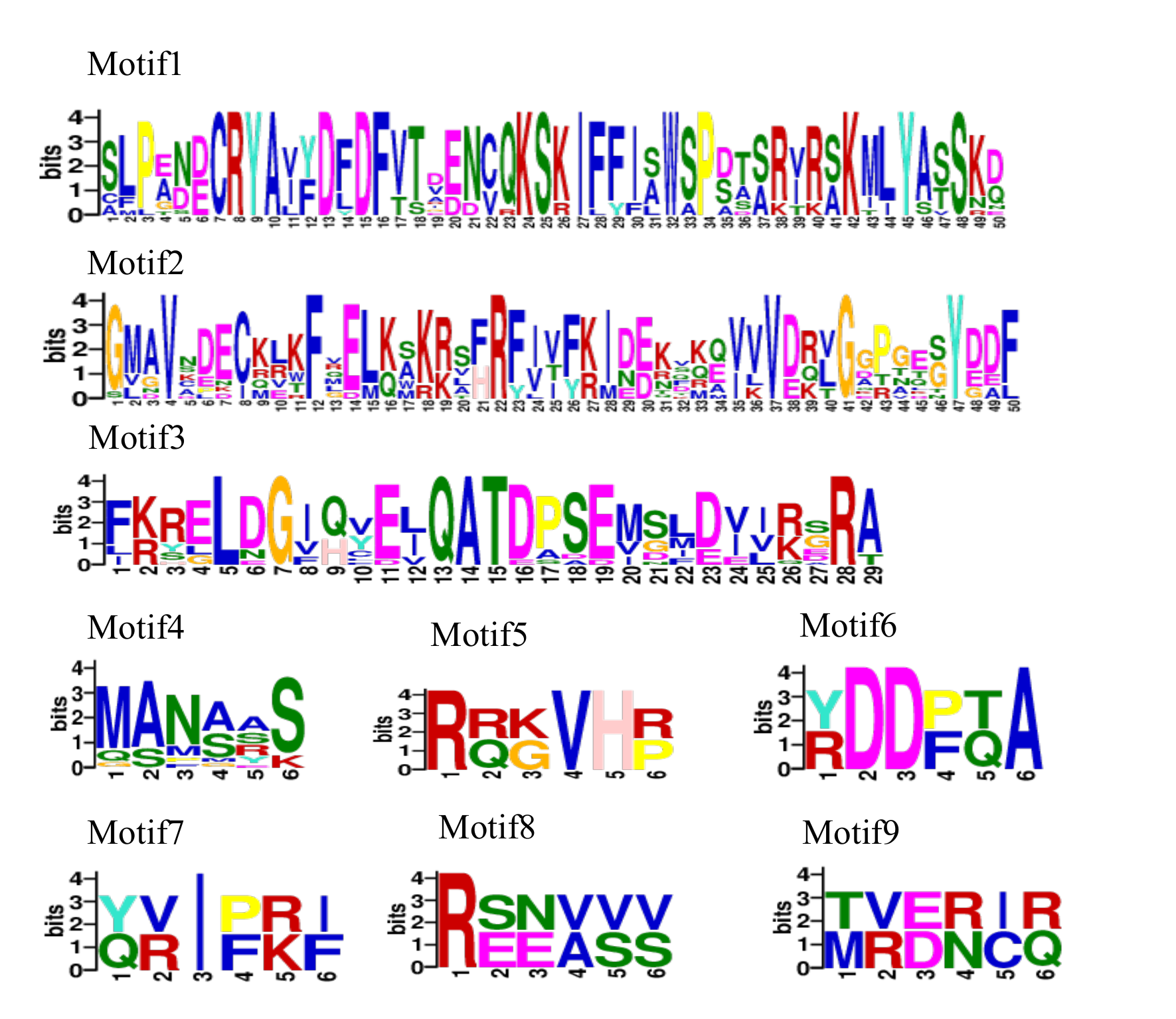

Supplement: Supplementary file 1 [file ijms-21-01751-s001.zip › Figure S1.tiff]

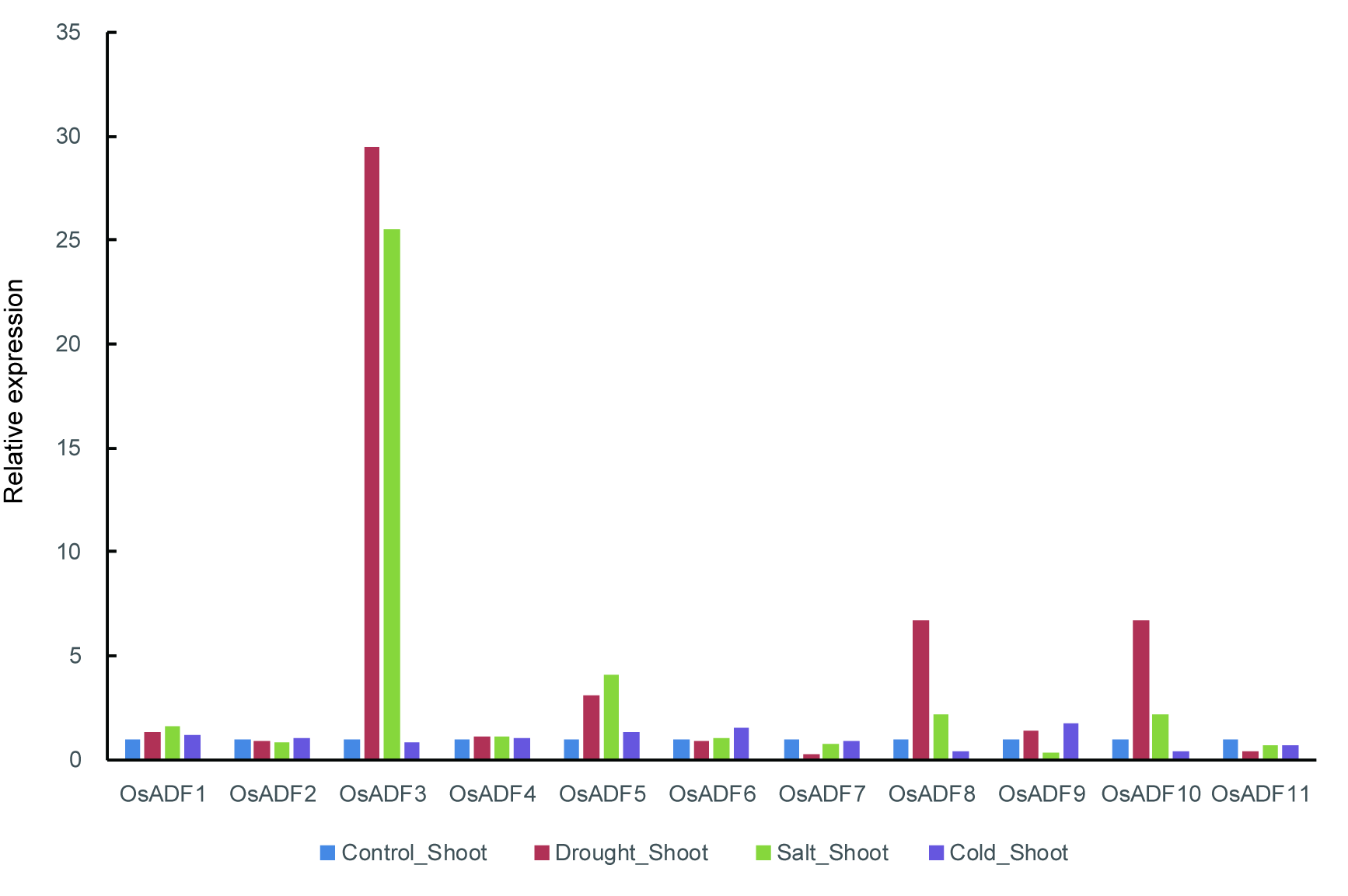

Supplement: Supplementary file 1 [file ijms-21-01751-s001.zip › Figure S2.tif]
